# What is the cross-sectional association of geospatially derived walkability with walking for leisure and transport?

**DOI:** 10.1371/journal.pone.0320202

**Published:** 2025-03-21

**Authors:** Adalberto A. S. Lopes, Larissa L. Lima, Amanda S. Magalhães, Amanda C. S. Andrade, Tiago Canelas, Louise Foley, Tolu Oni, Waleska T. Caiaffa

**Affiliations:** 1 Observatory for Urban Health in Belo Horizonte, Federal University of Minas Gerais, Brazil; 2 Center for Modeling Social Systems, Norwegian Research Centre, Kristiansand, Norway; 3 MRC Epidemiology Unit, University of Cambridge, England, United Kingdom.; Iran University of Medical Sciences, IRAN, ISLAMIC REPUBLIC OF

## Abstract

**Background:**

Built environments have been shown to shape active living behaviours, including walking. However, this literature is drawn predominantly from Europe and North America. This study aimed to create a geospatially derived city-wide walkability index and further investigate the association with walking in Belo Horizonte, Brazil.

**Methodology:**

A cross-sectional analysis was conducted using data from participants in the 2014-15 MOVE-SE study in Belo Horizonte. A walkability index was created at the census tract level, which included net residential density, land use mix, and street connectivity, using ArcGIS software. Walking for leisure and transportation was self-reported via the International Physical Activity Questionnaire. Covariates such as sociodemographic characteristics, health indicators, and neighbourhood context were measured. A multilevel negative binomial regression was employed, incorporating confounders across five combined models with sequential addition of covariate groups. All statistical analyses were conducted in R software with a significance threshold of 5%.

**Results:**

The study included 1,372 adults aged 18 years and older, with a female majority of 60.5%, a median age of 41, and 45.9% completed at most primary schooling. The family income for 63.7% ranged between one to three times the minimum wage. Self-rated health was considered good by 64.7% of participants, and the median Body Mass Index (BMI) was 26.2 kg/m2. Regarding neighbourhood context, the median length of residence was 15 years, per capita monthly income was US$175, and the average land slope was 8.2%. Participants reported a median of 180 minutes per week (interquartile range: 120 – 250) for walking for leisure and transportation. The median walkability index was -0.51 (interquartile range: -1.40 – 1.21). After adjusting for confounders, the final model indicated a positive association between the walkability index and walking for leisure (IRR: 1.33; CI_95%_:1.32-1.35; p < 0.001) and transportation (IRR: 1.22; CI_95%_:1.20-1.24; p < 0.001).

**Discussion:**

The findings demonstrate a positive association between higher levels of walkability and increased walking behaviours in various contexts. It underscores the importance of urban planning, design, and policy interventions tailored to local environments to promote walkability, reduce car dependency, and facilitate healthier lifestyles as part of everyday living.

## Background

Increasingly, urban spaces are recognized as important determinants of health [1]. Changing the social, natural, and physical environment in these spaces can promote healthy behaviour, especially in low-middle-income countries [2]. Even though each city has local demands and priorities [3], it is crucial to have a minimum of infrastructure, safety, and access to services so that people can insert healthy habits into their daily routines [4]. Environmental sustainability may make cities people-centred, enabling activity-friendly living [5–8], leading to a healthier population, thus, generating less cost to public health systems [9]. Nevertheless, isolated research indicators may not capture the complex health-environment interplay [10,11].

Urban attributes differently affect health outcomes [12]. Walkability, green spaces, and other amenities for health and wellbeing [13], high residential density, high density of public transport stops, presence of urban facilities, traffic safety, pedestrian infrastructure, the density of land use mix [5,6] the vicinity of parks and air quality are some of the features that have been associated with health behaviours or outcomes in urban dwellers, directly and indirectly impacting on the decrease in morbidities throughout life [14]. Physical activity opportunities are greatest in areas with connected streets and safe sidewalks [15], suggesting that walkable infrastructure promotes community health [16]. It indicates that contextual dimensions impacting physical activity must be considered in public policy promotion, incorporating people’s perceptions and features of the built, natural, and social environment where they live [17].

Geospatially derived walkability macroscale measures are utilized globally [18], affecting behaviours like sedentary lifestyles [19], mental health [20], and physical activity [21]. Frank et al. (2010) proposed a widely used walkability approach. Despite the considerable range of definitions, it can be described as how walk-friendly an urban place is, ensuring pedestrian attractiveness, comfort, and safety, with efficient access to varied, convenient, and desired destinations [22]. It harmonizes sustainable living with individual needs [23].

In addition to contributing 33-68% of total physical activity [24], active travel can benefit communities, especially in large cities, improving micro-mobility, and air quality, stimulating interaction between neighbours and access to local commerce, and reducing daily journey time [25]. However, while the prevalence of walking is 26% in the United Kingdom [26], as per the latest U.S. household travel survey data, with only 10% of all trips being done on foot, merely 14.3% of Americans achieve at least 10 minutes of walking, covering nearly 147 kilometres, in 37 hours, annually [27]. The median prevalence of walking in Latin America and the Caribbean is quite similar, with just 15% of the population undertaking any active travel [28]. For decades, city planning and management have been centred more on the automobile than pedestrians, providing a system that makes driving convenient and presents obstacles to walking, such as long distances to access destinations, poor quality sidewalks, and urban insecurity. Nevertheless, a paradigm shift is urgent, as the evidence demonstrates that people who live in the most active-friendly neighbourhoods are involved in up to 90 minutes more walking per week than those whose environments do not support active living [29].

Addressing the global yet diverse urban challenges requires tailored solutions [17,30]. In Brazil, unstable walking levels in cities reflect environmental and policy issues [31]. Therefore, to guide policymakers in developing strategies to promote active mobility policies, this study aimed to create a city-wide walkability index based on geospatially derived urban environment macroscale features and further investigate the association of the walkability index with walking for leisure and transportation in Belo Horizonte, Brazil.

## Methods

### Study design and area

This cross-sectional study was led by the Observatory for Urban Health in Belo Horizonte, from the Federal University of Minas Gerais (OSUBH/UFMG), Brazil, in collaboration with the Global Diet and Activity Research (GDAR) network. The datasets used are mixed primary and secondary sources in Belo Horizonte, the capital of the Minas Gerais state, located in the Southeast region of Brazil. The city has 2.5 million inhabitants and is Brazil’s sixth-largest urban area and the fifth-largest Gross Domestic Product among Brazilian cities [32]. The Municipal Human Development Index (MHDI) for the municipality is 0.810, placing it in the very high human development category. This is due to strong performance in longevity (0.856), income (0.841), and education (0.737). However, the municipality has notable income inequality, as indicated by a Gini index of 0.54 (IBGE 2010).

### Sampling, data gathering, and ethical issues

The primary data came from a cross-sectional study conducted by OSUBH/UFMG carried out between November 2014 and March 2015, entitled MOVE-SE, Lifestyles and Health Project. The home-based health survey included as sample, resident population of ≥ 18 years old in a geographical area served by a health promotion programme. In a face-to-face interview, the data were gathered using a standardized questionnaire that assessed topics related to the individual, home-related and neighbourhood characteristics, as well as aspects related to participation in the program and health service use. The project was approved by the Research Ethics Committee of the Federal University of Minas Gerais (process number CAAE: 26152814.2.0000.5149 of May 8^th^, 2014), in which all participants signed an informed written consent form. More details may be obtained in a previous publication [33,34].

### Construction of a walkability index

**Fig 1:**
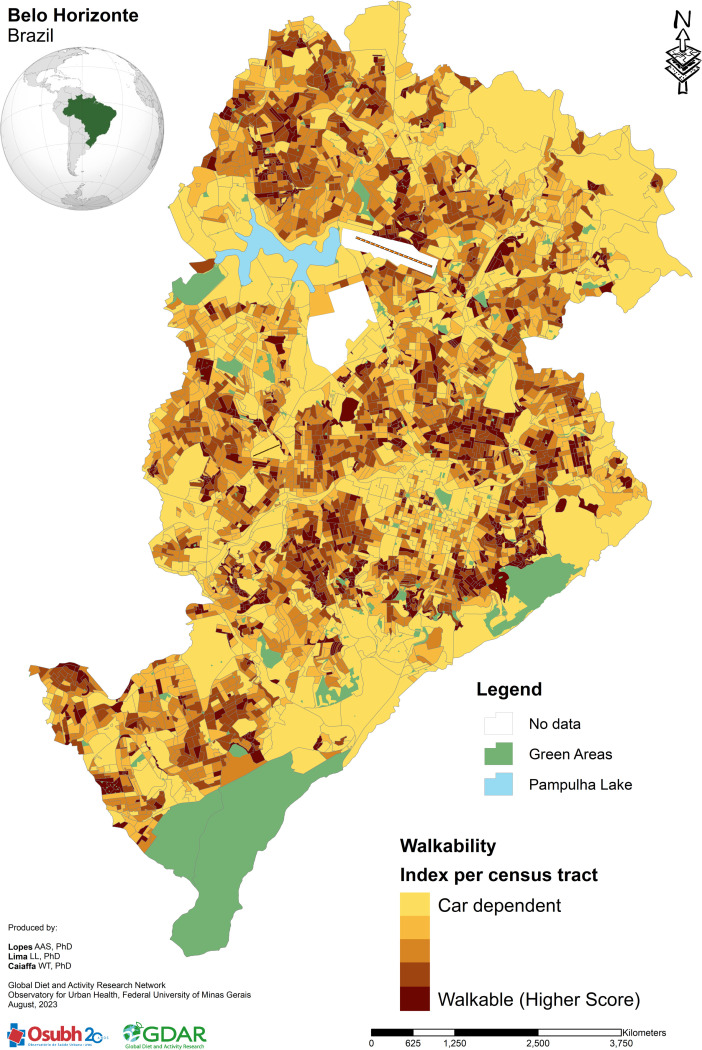
Map distribution of walkability index per census tract in Belo Horizonte, Brazil.

To navigate the complex array of urban environment indicators that impact health, a macroscale index measure was employed [11]. The walkability index was built comprising three combined indicators: land use mix, intersection density, and net residential density, converted into a standard deviation unit using the Z-score, allowing for comparison on a common scale [35]. The geographical unit of analysis was the census tract – a continuous area delineated for effective data gathering, characterized by its manageability in size and population [32]. The ArcGIS software (v.10.5 for Desktop, Environment Systems Research Institute, Redlands-CA, USA) was used to harmonize and analyse data, which are publicly available on the website of different official sources, such as Municipality of Belo Horizonte (BHMap), Brazilian Institute of Geography and Statistics (IBGE), National Classification of Economic Activities (CNAE), and Open Street Map (OSM). For ease of understanding, Fig 1 illustrates the spatial distribution of the index across the city. It labels the extremities of the index spectrum as either “car-dependent” or “walkable” [14]. The “walkable” category represents areas that scored highest on the index. More detailed information is at the supplementary material C1 and F1.

### Exposure variable

Once the city-wide walkability index was established, individual MOVE-SE project participants were assigned an index score based on their residence’s census tract location. This index, treated as continuous data following the literature [36], was applied to 246 out of 3,933 census tracts.

### Outcome variables

As part of the primary dataset, self-reported physical activity, collected using the long version of the International Physical Activity Questionnaire (IPAQ), was obtained from the MOVE-SE project [33,34]. They included frequency (days/week) and duration (minutes/day) of walking for leisure and transportation [37–39]. The questionnaire used was based on the structure and content of a Brazilian national surveillance, which has validity and reproducibility established [40]. Because the data presented a non-normal distribution, the outcomes were kept continuous for the analysis, considering minutes per week as the unit of measurement [41].

### Covariates

Individual covariates were used to describe the sample and adjust the analytical models. The sociodemographic data were: a) sex, male and female; b) age, in years; c) schooling, classified as “up to primary”, “secondary”, and “university”; d) family income, based on the minimum wage (MW) in Brazilian Real (R$) set in R$ 724,00 (in 2014), and classified “up to 1 MW”, “between 2-3 MW” and “more than 3 MW”. The health indicators selected were self-rated health classified as “good”, “regular” and “poor” [42] and the nutritional status was the body mass index (BMI), the ratio between weight in kilograms and height in square meters [43]. The neighbourhood context considered the length of residence, the time the participant lives at the same address; the income *per capita* (R$), and the land slope percentage using geospatial data, considering the median values for the census tract where each participant’s residence is placed.

### Data analysis

Based on the walking for leisure and transportation high zero proportion, the most popular (*log*) or complex (*square root*) transformations for a non-normal continuous outcome were unfeasible (Supplement F2 and F3). Considering the dataset’s characteristics and the study design, the sample weight and a generalized linear mixed models (GLMM) were applied. A multilevel negative binomial regression (NBR) was selected to investigate the association between continuous walkability index and walking for leisure and transportation as the most suitable GLMM analytical model after considering mainly the Akaike Information Criterion (AIC) and the Dispersion Parameter values (Supplement T1). The application of NBR results in the Incidence Rate Ratio (IRR), which reflects in cross-sectional studies an association between exposures and outcomes at a single point in time, not allowing causality or temporal relationships. A conceptual model structured in a Directed Acyclic Graph guided the minimal sufficient adjustment sets for estimating the total effect of the exposure on outcomes, which included the confounders in five models combined, with covariates groups inputted one by one (Supplement F4). All analyses were run using R software (v.2023.03.1 build 446, PBC) for macOS, and statistical significance was set at 5%.

## Results

### The city-wide walkability index

Belo Horizonte has 3,933 census tracts, with a median of 0.049 km^2^ of area, 11,651 inhabitants/km^2^, R$743.65 Brazilian Real – representing an urban contextual income, and a land slope of 7.6%. The index comprised three indicators: land use mix (median: -0.30), intersection density (median: -0.18), and net residential density (median: 0.03). The median of walkability was -0.60 (IQR: -1.60 – 0.49), ranging between -5.37 to 175.69, where larger values represent a higher level of the index, as is shown in Table 1.

**Table 1:** Descriptive statistics of census tract the geospatial-based macroscale indicators composition, and the walkability index in Belo Horizonte, Brazil. (n = 3,933).

Variable	Unit of measurement	Median (IQR)	Min-Max
Census tract	Area (km^2^)	0.04 (0.02 – 0.07)	<0.001 – 4.930
Populational density	Inhabitants/km^2^	11,651 (8,158 – 17,267)	0 – 420,036
Contextual income	Brazilian real (R$)	743.65 (496.78 – 1,517.94)	0.00 – 22,955.85
Land slope	%	7.6 (5.3 – 11.2)	0.0 – 40.0
			
Land use			
*Retail*	Z-score	-0.05 (-0.06 – -0.04)	-0.08 – 38.78
*Entertainment*	Z-score	-0.11 (-0.11 – -0.06)	-0.12 – 52.09
*Food-related*	Z-score	-0.05 (-0.05 – -0.04)	-0.06 – 40.01
*Civic/Institutional*	Z-score	-0.04 (-0.05 – -0.02)	-0.06 – 47.51
*Office*	Z-score	-0.07 (-0.07 – -0.06)	-0.09 – 27.92
*Public recreation*	Z-score	-0.02 (-0.02 – -0.02)	-0.02 – 59.99
*Private recreation*	Z-score	-0.03 (-0.03 – -0.03)	-0.03 – 47.15
Land use mix	Entropy^†^	-0.30 (-0.70 – -0.12)	0.03 – 1.00
Intersection density	Z-score	-0.18 (-0.57 – 0.32)	-1.44 – 8.86
Net residential density	Z-score	0.03 (-0.37 – 0.21)	-0.94 – 22.10
**Walkability** ^‡^	**Index**	**-0.60 (-1.60 – 0.49)**	**-5.37 – 175.69**

IQR*: Interquartile range;* ***Min-Max****: Minimum and maximum;* ***†****: The concept of entropy was introduced by Claude Shannon (1948);* ***‡****: The walkability index was developed according to Frank (2010).*

### The survey sampling

**Fig 2:**
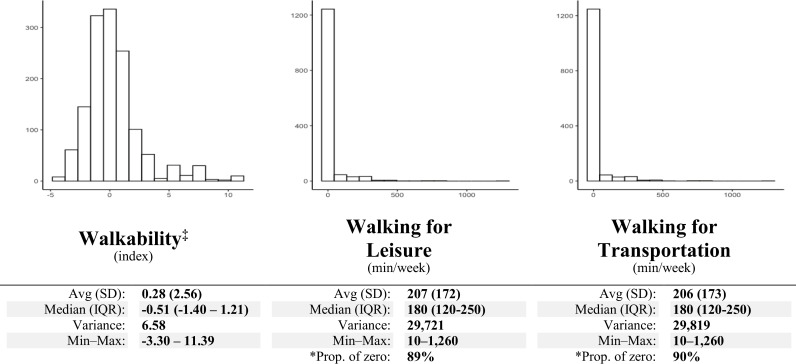
Descriptive statistics of the exposure and outcomes variables in Belo Horizonte, Brazil. (n = 1,372). Avg: Average; SD: Standard deviation; IQR: Interquartile range; Min-Max: Minimum-maximum; ‡: Developed according to Frank (2010), representing in this study the census tract (246/3,933) in which each participant’ residence is placed; *: Due to the IPAQ use, which counts undertaking from 10 minutes, the proportion of zero represents those participants who did not undertaking any physical activity.

With 1,372 adults (≥18 years old) from Belo Horizonte, the median walkability based on the participant’s residence’s census tract (n = 246) location was -0.51 (IQR: -1.40 – 1.21) ranging from -3.30 to 11.39, where higher scores indicate greater walkability. Furthermore, the median walking for leisure and transportation was 180 min/week (IQR: 120 – 250), while the 0 min/week participants were 89% and 90%, respectively (Fig 2).

Most of the sample was composed of women (60.5%), with a median age of 41 years old, 45.9% educated up to primary schooling, and 63.7% with family income between 1 to 3 minimum wages. The majority reported good self-rated health (64.7%), and the median BMI was 26.2 kg/m^2^, classified as overweight. For the neighbourhood context variables, people reported living in the same place for a median of 15 years; regarding the geospatial-based macroscale measures, the median income *per capita* was below minimum wage at US$ 175 (for the period of participant’s data gathering, as per IPEA - Institute of Applied Economic Research of Brazil) and the land slope was 8.2%. Additionally, the crude associations were similar and in the same direction — the incidence rate ratio of both physical activity domains was higher in males, older, more educated, and wealthier participants than their counterparts. The same reading can be observed for the health indicators, with better self-rated health and higher BMI in those who perform walking for leisure and transportation. Longer residence time and higher income *per capita* were also associated with the outcomes, while no significant association with land slope was observed, as is shown in Table 2.

**Table 2:** Descriptive analysis of the sample and multilevel negative binomial regression between covariates, exposure & walking for leisure and transportation in Belo Horizonte, Brazil. (n = 1,372).

Covariate	Sample features	Physical activity domain	
**Walking for** **Leisure** 180 min/week **(IQR: 120 – 250)**	**Walking for Transportation** 180 min/week **(IQR: 120 – 250)**
n (%)^†^ | Median (IQR)^‡^	IRR (CI_95%_)^*^	IRR (CI_95%_)^*^
*Sociodemographic*			
Sex male (*female* ref.)	542 (39.5)^†^	1.69 (1.59-1.80)	1.81 (1.70-1.93)
Age (*years*)	41.0 (28.0-54.0)^‡^	1.03 (1.02-1.04)	1.03 (1.02-1.04)
Schooling			
*Up to primary (ref.)*	633 (46.1)^†^	1	1
*Secondary*	581 (42.3)^†^	1.41 (1.33-1.49)	1.47 (1.39-1.56)
*University*	158 (11.5)^†^	1.74 (1.59-1.91)	1.86 (1.70-2.04)
Family income ^[a]^			
*Up to 1 MW (ref.)*	67 (4.2)^†^	1	1
*Between 1-3 MW*	821 (63.7)^†^	1.67 (1.45-1.92)	1.67 (1.45-1.93)
*More than 3 MW*	451 (32.1)^†^	2.16 (1.88-2.49)	2.05 (1.78-2.37)
*Health indicator*			
Self-rated health			
*Good (ref.)*	938 (64.7)^†^	1	1
*Regular*	342 (25.9)^†^	0.57 (0.53-0.60)	0.57 (0.53-0.61)
*Poor*	92 (9.4)^†^	0.05 (0.04-0.06)	0.05 (0.04-0.06)
Nutritional status (BMI) ^[b]^	26.2 (23.0-30.2)^‡^	1.13 (1.12-1.14)	1.14 (1.13-1.15)
*Neighbourhood context*			
Length of residence (*years*) ^[c]^	15.0 (4.0-28.0)^‡^	1.02 (1.01-1.03)	1.03 (1.02-1.04)
Income *per capita* (*R$)*^§^	584.6 (421.4-787.5)^‡^	1.28 (1.17-1.39)	1.29 (1.18-1.41)
Land slope (%)^§^	8.2 (5.8-11.5)^‡^	0.98 (0.92-1.03)	0.98 (0.93-1.04)

***IQR****: Interquartile range;* ***MW****: Minimum wage set in R$724,00;* ***BMI****: Body Mass Index in kg/m*^*2*^; ***IRR****: Incidence rate ratio;* ***CI***_*95%*_*: Confidence interval;*

† *: the categorical variables, reported by absolute and relative frequency;*

‡ *: the continuous variables, reported by measures of central tendency and interquartile range;*

* *: Considered only one covariate at a time, inputted one by one, furthermore, all multilevel analysis used a random intercept for census tract and regarded the sample weight; [a]: 33 missing values; [b]: 14 missing values; [c]: 9 missing values;*

§ *: Geospatially derived urban macroscale measures (census tract).*

**Fig 3:**
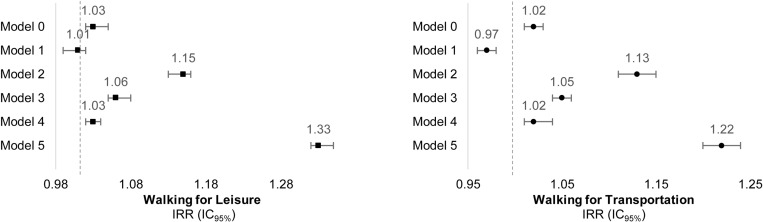
Multilevel negative binomial regression models of walkability index & walking for leisure and transportation in Belo Horizonte, Brazil. (n = 1,372). IRR: Incidence rate ratio; CI95%: Confidence interval; Models: All multilevel analysis used a random intercept for census tract and regarded the sample weight; Model 0: Crude analysis; Model 1: Adjusted by individual sociodemographic; Model 2: Adjusted by health indicators; Model 3: Adjusted by neighbourhood contextual; Model 4: Adjusted by the interaction of income and land slope, geospatially derived urban macroscale measures (census tract); Model 5: Models of 1 to 4 combined.

All models (Supplement T2) were positively associated between the walkability index and walking outcomes, except model 1 for leisure [where the result demonstrates no statistical significance] and transportation [in which the association was inverse]. Both included only individual sociodemographic covariates. After adjusting for confounders, the final models resulted in a greater association between the walkability index and walking for leisure (IRR: 1.33; CI_95%_:1.32-1.35; p < 0.001) and transportation (IRR: 1.22; CI_95%_:1.20-1.24; p < 0.001), as is shown in Fig 3.

## Discussion

This study derived a city-wide walkability index for Belo Horizonte, Brazil, utilizing geospatially derived macroscale features of the urban environment. It aims to inform policymakers on formulating active mobility strategies. Key strengths of the study are highlighted, as the methodology draws upon well-established techniques literature-based for evaluating walkability [35], integrates a robust study design tailored to a Latin American urban context [33,34], and employs suitable statistical modelling techniques to address dataset peculiarities [36,41].

Urban models promoting walkable cities are gaining traction globally. The initial finding of this study reveals that, based on geospatially derived metrics such as land use mix, intersection density, and net residential density, Belo Horizonte exhibits a moderate walkability index, suggesting a tendency towards car dependency rather than walkability. In part, this phenomenon can be attributed to the city’s zoning, where residential areas are segregated from hubs of daily activities like work and study, despite well-connected streets. The city’s latest master plan underscores the importance of multi-sectoral integration to address the Sustainable Development Goals (SDGs), particularly Goal 11, which pertains to sustainable cities and communities. Undoubtedly, the challenges are significant. Among the priorities is reducing reliance on the city centre and individual motorized transport. This entails improving urban infrastructure, enhancing the permeability of land, promoting densification centred around public transport, establishing facilities to encourage active urban mobility, and preserving the natural environment [44].

Despite the concentration of businesses in the central area, mixed land use or non-residential purposes have expanded, even if only modestly. This underscores the importance of diverse functions within neighbourhoods, enabling residents to access services and commerce nearby, thus reducing lengthy commutes, alleviating traffic congestion, and enhancing the daily lives of citizens. In fact, much like other major Brazilian cities, Belo Horizonte was shaped to prioritize mobility through individual motorized vehicles [45]. This leads to consequences for pedestrian movement, especially for those who use walking as a transportation mode. To address this situation, new proposals and projects have been developed to value public spaces and pedestrians. The Belo Horizonte Mobility Plan emphasizes the need for measures that promote pedestrian walking, such as actions to discourage car usage and investments in pedestrian infrastructure [46]. However, despite structural advancements, pedestrian walking is still undervalued in urban planning, even though it is the city’s most utilized mode of transportation [47]. Another pressing concern is the adverse effects of heavy rainfall. It is estimated that 144 areas in Belo Horizonte are at high risk of flooding, with a projected 32% increase in issues stemming from intense rains. These include mortality, property damage, heightened susceptibility to infectious diseases like dengue, and impacts on active travel throughout the city [48].

Broadening the geographical applicability of these findings beyond Europe and North America [49,50], this study’s findings corroborate existing literature affirming a positive association between walkability and walking. The outcome aligns with a comprehensive scoping review by Dixon et al [51], where they described a positive association between walkability and physical activity in 83% of the reviews. Such results are not surprising as it is consistent with the purpose of walkability indices, which are designed to evaluate environments conducive to walking [35]. As this study’s findings also reveal, walkability has a multi-dimensional impact as it not only influences leisure-time physical activity but also has a broader impact on transportation-related walking. Urban models promoting walkable cities are gaining traction globally. These discoveries are congruous with the international literature, which indicates that walkability and/or its components are positively associated with active transport and total physical activity [51–53]. Indeed, this is one of the most consistent findings in this field.

Urban health is a complex system in which several environmental attributes act concurrently [11,54]. This approach, besides providing a more realistic view of cities, allows a systematic contextual analysis of how walkable a place can be [12,13]. Especially when it is comprehensible that in several urban contexts, regardless of infrastructure, walking is not an individual’s choice but a necessity [17]. Walkability parallels mixed land uses [55], with access through street pedestrian-level to the diversity of destinations such as service, commerce, leisure, study, and workplace nearby. Thus, it seems a preponderant factor that a city would be compactness [56] and connectedness [15] to provide environmental benefits to the population, stimulating a walking-friendly place and reducing organically car dependency [57]. Concepts of 15 or 20-minute cities appears in the literature as great to favour urban scenario that reclaims public spaces for people [58,59].

Limitations of this study are highlighted to facilitate an adequate interpretation of the findings. Parsimony due to the study design, once the association is identified, cannot be generalized to the whole city. In fact, there is an influence on living near or even attending a health academy program centre [60]. However, the sample’s walkability index was able to capture the city-wide walkability index variability. Although the most widely used instrument in population-based studies, the physical activity tool for measuring walking has a low sensitivity over distances shorter than 10 minutes of travel [37,38]. Furthermore, the environmental indicators do not consider microscale street pedestrian level nor neighbourhoods’ citizen perception, such as sidewalk quality, presence of urban furniture, road signage, traffic, and crime safety, as they may be relevant aspects of decision-making to walk or drive [61,62]. Disregarding potential changes over time, based on a cross-sectional approach, geospatially derived macroscale measures, despite allowing a wide-ranging territorial analysis, present data from a different period than the outcomes. Additionally, the walkability index creation did not consider parsimonious methods to determine which and how many indicators would be appropriate for the contextual reality of the city [63]. However, it allows comparability with most studies worldwide related to the topic [18], which constructs the index from this set of geographical scale indicators [35]. Due to the steep slope characteristic of the city, this indicator should be part of the index, considered a local peculiarity [64,65]. Nonetheless, the slope was included in an analytical model as it is considered relevant for the finding’s interpretation.

Suggestions for future investigations are highlighted to guide new research questions. An agent-based model should be integrated into the walkability index monitoring over time [66], simulating urban scenarios to test the effectiveness of implementing or changing city features [67]. Combine mixed methods to assess the environment’s macro and microscale indicators, incorporating built, natural, and social city’ attributes beyond increased physical activity measures through accelerometery and their urban contextual using global positioning system-wearable devices to identify destinations, durations, and distances objectively-derived [68]. Finally, the longitudinal and experimental study design should be prioritized, and the population and stakeholders should be jointly considered to identify the relevant and feasible indicators to compose the walkability city index.

In conclusion, considering the city as a complex and multi-component system, our findings suggest that increasing the walkability of neighbourhoods can be an effective strategy for increasing active living behaviour. Furthermore, specific aspects of the planning, design and implementation of policies and interventions to create more walk-friendly places should be tailored to local contexts, taking people into account to decrease car-dependence and provide opportunities to improve the health of citizens as part of their daily routine.

## Supporting information

Supporting Materials Containing supporting figures 1-4 and supporting table 1-2. (PDF)

## References

[1] Kleinert S, Horton R. Urban design: an important future force for health and wellbeing. Lancet. 2016;388(10062):2848–50. doi: 10.1016/S0140-6736(16)31578-1 27671666

[2] Panter J, Guell C, Prins R, Ogilvie D. Physical activity and the environment: conceptual review and framework for intervention research. Int J Behav Nutr Phys Act. 2017;14(1):156. doi: 10.1186/s12966-017-0610-z 29141646 PMC5688667

[3] Pratt M, Ramirez Varela A, Kohl HWB, Klepac Pogrmilovic B, Pedišić Ž, Sallis JF. Plan globally and act locally for physical activity?. J Phys Act Health. 2021;18(10):1157–8. doi: 10.1123/jpah.2021-0471 34412034

[4] Salvo D, Garcia L, Reis RS, Stankov I, Goel R, Schipperijn J, et al. Physical activity promotion and the United Nations sustainable development goals: building synergies to maximize impact. J Phys Act Health. 2021;18(10):1163–80. doi: 10.1123/jpah.2021-0413 34257157

[5] Cerin E, Sallis JF, Salvo D, Hinckson E, Conway TL, Owen N, et al. Determining thresholds for spatial urban design and transport features that support walking to create healthy and sustainable cities: findings from the IPEN Adult study. Lancet Glob Health. 2022;10(6):e895–906. doi: 10.1016/S2214-109X(22)00068-7 35561724 PMC9731787

[6] Lowe M, Adlakha D, Sallis JF, Salvo D, Cerin E, Moudon AV, et al. City planning policies to support health and sustainability: an international comparison of policy indicators for 25 cities. Lancet Glob Health. 2022;10(6):e882–94. doi: 10.1016/S2214-109X(22)00069-9 35561723 PMC9906636

[7] Giles-Corti B, Moudon AV, Lowe M, Cerin E, Boeing G, Frumkin H, et al. What next? Expanding our view of city planning and global health, and implementing and monitoring evidence-informed policy. Lancet Glob Health. 2022;10(6):e919–26. doi: 10.1016/S2214-109X(22)00066-3 35561726

[8] Shilton T, Bauman A, Beger B, Chalkley A, Champagne B, Elings-Pers M, et al. More people, more active, more often for heart health - taking action on physical activity. Glob Heart. 2024;19(1):42. doi: 10.5334/gh.1308 38708404 PMC11067976

[9] Santos AC, Willumsen J, Meheus F, Ilbawi A, Bull FC. The cost of inaction on physical inactivity to public health-care systems: a population-attributable fraction analysis. Lancet Glob Health. 2023;11(1):e32–9. doi: 10.1016/S2214-109X(22)00464-8 36480931 PMC9748301

[10] Milton K, Cavill N, Chalkley A, Foster C, Gomersall S, Hagstromer M, et al. Eight investments that work for physical activity. J Phys Act Health. 2021;18(6):625–30. doi: 10.1123/jpah.2021-0112 33984836

[11] Rybski D, González MC. Cities as complex systems-Collection overview. PLoS One. 2022;17(2):e0262964. doi: 10.1371/journal.pone.0262964 35213566 PMC8880833

[12] Elshahat S, O’Rorke M, Adlakha D. Built environment correlates of physical activity in low- and middle-income countries: A systematic review. PLoS One. 2020;15(3):e0230454. doi: 10.1371/journal.pone.0230454 32182278 PMC7077823

[13] Ige-Elegbede J, Pilkington P, Orme J, Williams B, Prestwood E, Black D, et al. Designing healthier neighbourhoods: a systematic review of the impact of the neighbourhood design on health and wellbeing. Cities Health. 2020;6(5):1004–19. doi: 10.1080/23748834.2020.1799173 36618774 PMC9810039

[14] Frank LD, Adhikari B, White KR, Dummer T, Sandhu J, Demlow E, et al. Chronic disease and where you live: Built and natural environment relationships with physical activity, obesity, and diabetes. Environ Int. 2022;158:106959. doi: 10.1016/j.envint.2021.106959 34768046

[15] Zhang Y, van Dijk T, Wagenaar C. How the built environment promotes residents’ physical activity: the importance of a holistic people-centered perspective. Int J Environ Res Public Health. 2022;19(9):5595. doi: 10.3390/ijerph19095595 35564990 PMC9101533

[16] Bird EL, Ige JO, Pilkington P, Pinto A, Petrokofsky C, Burgess-Allen J. Built and natural environment planning principles for promoting health: an umbrella review. BMC Public Health. 2018;18(1):930. doi: 10.1186/s12889-018-5870-2 30055594 PMC6064105

[17] Salvo D, Jáuregui A, Adlakha D, Sarmiento OL, Reis RS. When moving is the only option: the role of necessity versus choice for understanding and promoting physical activity in low- and middle-income countries. Annu Rev Public Health. 2023;44::151–69. doi: 10.1146/annurev-publhealth-071321-042211 36525957

[18] Sallis JF, Cerin E, Kerr J, Adams MA, Sugiyama T, Christiansen LB, et al. Built environment, physical activity, and obesity: findings from the International Physical Activity and Environment Network (IPEN) Adult Study. Annu Rev Public Health. 2020;41:119–39. doi: 10.1146/annurev-publhealth-040218-043657 32237990

[19] Rescarolli M, Neto FT de P, Lopes AADS, Justina MDD, da Silva AQA, d’Orsi E, et al. Is walk score associated with physical activity and screen time in brazilian older adults?. J Aging Phys Act. 2023;31(6):956–64. doi: 10.1123/japa.2022-0165 37263594

[20] Siqueira Junior J de A, Lopes AADS, Godtsfriedt CES, Justina MDD, de Paiva KM, d’Orsi E, et al. Neighbourhood walkability and mental health in older adults: A cross-sectional analysis from EpiFloripa Aging Study. Front Aging. 2022;3:915292. doi: 10.3389/fragi.2022.915292 36523860 PMC9745083

[21] Siqueira Reis R, Hino AAF, Ricardo Rech C, Kerr J, Curi Hallal P. Walkability and physical activity: findings from Curitiba, Brazil. Am J Prev Med. 2013;45(3):269–75. doi: 10.1016/j.amepre.2013.04.020 23953352 PMC3748398

[22] Tobin M, Hajna S, Orychock K, Ross N, DeVries M, Villeneuve PJ, et al. Rethinking walkability and developing a conceptual definition of active living environments to guide research and practice. BMC Public Health. 2022;22(1):450. doi: 10.1186/s12889-022-12747-3 35255841 PMC8900439

[23] Baobeid A, Koç M, Al-Ghamdi SG. Walkability and its relationships with health, sustainability, and livability: elements of physical environment and evaluation frameworks. Front Built Environ. 2021;7. doi: 10.3389/fbuil.2021.721218

[24] Prince SA, Lancione S, Lang JJ, Amankwah N, de Groh M, Garcia AJ, et al. Are people who use active modes of transportation more physically active? An overview of reviews across the life course. Transp Rev. 2021;42(5):645–71. doi: 10.1080/01441647.2021.2004262

[25] Ferretto L, Bruzzone F, Nocera S. Pathways to active mobility planning. Res Transport Econ. 2021;86:101027. doi: 10.1016/j.retrec.2020.101027

[26] Buehler R, Pucher J. Overview of walking rates, walking safety, and government policies to encourage more and safer walking in Europe and North America. Sustainability. 2023;15(7):5719. doi: 10.3390/su15075719

[27] Buehler R, Pucher J, Bauman A. Physical activity from walking and cycling for daily travel in the United States, 2001–2017: Demographic, socioeconomic, and geographic variation. J Transp Health. 2020;16:100811. doi: 10.1016/j.jth.2019.100811

[28] de Sá TH, de Rezende LFM, Borges MC, Nakamura PM, Anapolsky S, Parra D, et al. Prevalence of active transportation among adults in Latin America and the Caribbean: a systematic review of population-based studies. Rev Panam Salud Publica. 2017;41:e35. doi: 10.26633/RPSP.2017.35 31363356 PMC6614750

[29] Sallis JF, Bull F, Burdett R, Frank LD, Griffiths P, Giles-Corti B, et al. Use of science to guide city planning policy and practice: how to achieve healthy and sustainable future cities. Lancet. 2016;388(10062):2936–47. doi: 10.1016/S0140-6736(16)30068-X 27671670

[30] Murphy J, Milton K, Mclaughlin M, Shilton T, McLoughlin GM, Reece LJ, et al. Advocating for implementation of the global action plan on physical activity: challenges and support requirements. J Phys Act Health. 2022;20(1):10–9. doi: 10.1123/jpah.2022-0357 36476969

[31] Bastone A de C, Moreira B de S, Vasconcelos KS de S, Magalhães AS, Coelho DM, Silva JI da, et al. Time trends of physical activity for leisure and transportation in the Brazilian adult population: results from Vigitel, 2010-2019. Cad Saude Publica. 2022;38(10):e00057222. doi: 10.1590/0102-311XEN057222 36449841

[32] IBGE. Brazilian Population Census 2010. Brazilian Inst Geogr Stat (Instituto Bras Geogr e Estatística). 2010.

[33] Lopes MS, Caiaffa WT, Andrade AC de S, Malta DC, Barber S, Friche AA de L. Disparities in food consumption between economically segregated urban neighbourhoods. Public Health Nutr. 2020;23(3):525–37. doi: 10.1017/S1368980019003501 31839024 PMC10200452

[34] Magalhães AS, Moreira B de S, Costa DA da S, Andrade AC de S, Caiaffa WT. Association of mammography with sociodemographic and care factors in residents of Belo Horizonte, MG, Brazil. Mastology. 2020;30:1–9. doi: 10.29289/25945394202020200011

[35] Frank LD, Sallis JF, Saelens BE, Leary L, Cain K, Conway TL, et al. The development of a walkability index: application to the Neighborhood Quality of Life Study. Br J Sports Med. 2010;44(13):924–33. doi: 10.1136/bjsm.2009.058701 19406732

[36] Lamb KE, White SR. Categorisation of built environment characteristics: the trouble with tertiles. Int J Behav Nutr Phys Act. 2015;12:19. doi: 10.1186/s12966-015-0181-9 25889014 PMC4335683

[37] Craig CL, Marshall AL, Sjöström M, Bauman AE, Booth ML, Ainsworth BE, et al. International physical activity questionnaire: 12-country reliability and validity. Med Sci Sports Exerc. 2003;35(8):1381–95. doi: 10.1249/01.MSS.0000078924.61453.FB 12900694

[38] Hallal PC, Gomez LF, Parra DC, Lobelo F, Mosquera J, Florindo AA, et al. Lessons learned after 10 years of IPAQ use in Brazil and Colombia. J Phys Act Health. 2010;7 Suppl 2:S259-64. doi: 10.1123/jpah.7.s2.s259 20702914

[39] Bull FC, Al-Ansari SS, Biddle S, Borodulin K, Buman MP, Cardon G, et al. World Health Organization 2020 guidelines on physical activity and sedentary behaviour. Br J Sports Med. 2020;54(24):1451–62. doi: 10.1136/bjsports-2020-102955 33239350 PMC7719906

[40] Moreira AD, Claro RM, Felisbino-Mendes MS, Velasquez-Melendez G. Validity and reliability of a telephone survey of physical activity in Brazil. Rev Bras Epidemiol. 2017;20(1):136–46. doi: 10.1590/1980-5497201700010012 28513801

[41] Akram M, Cerin E, Lamb KE, White SR. Modelling count, bounded and skewed continuous outcomes in physical activity research: beyond linear regression models. Int J Behav Nutr Phys Act. 2023;20(1):57. doi: 10.1186/s12966-023-01460-y 37147664 PMC10163772

[42] Pucci G, Reis RS, Rech CR, Hallal PC. Quality of life and physical activity among adults: population-based study in Brazilian adults. Qual Life Res. 2012;21(9):1537–43. doi: 10.1007/s11136-011-0083-5 22362520

[43] WHO. World Health Organization: A healthy lifestyle - WHO recommendations. In: Online [Internet]. 2010. Available: https://www.who.int/europe/news-room/fact-sheets/item/a-healthy-lifestyle---who-recommendations A

[44] Espindola I, Ribeiro W. Cities and climate change: challenges to Brazilian municipal Master Plans. Cad Metropóle. 2020;22(1):365–94. doi: 10.1590/2236-9996.2020-4802

[45] Melo T da S, Mota JVL, Silveira NDB e, Andrade ARS de, Peres MCL, Oliveira MLT de, et al. Combining ecological knowledge with Brazilian urban zoning planning. urbe, Rev Bras Gest Urbana. 2020;12:1-15. doi: 10.1590/2175-3369.012.e20190135

[46] PBH. PlanMob-BH: Plano de Mobilidade Urbana de Belo Horizonte. Belo Horizonte; 2012.

[47] BHTRANS. Balanço Anual da Mobilidade Urbana de Belo Horizonte. 2019.

[48] Dalagnol R, Gramcianinov CB, Crespo NM, Luiz R, Chiquetto JB, Marques MTA, et al. Extreme rainfall and its impacts in the Brazilian Minas Gerais state in January 2020: Can we blame climate change?. Climate Resilience. 2021;1(1):. doi: 10.1002/cli2.15

[49] Van Dyck D, Cardon G, Deforche B, Sallis JF, Owen N, De Bourdeaudhuij I. Neighborhood SES and walkability are related to physical activity behavior in Belgian adults. Prev Med. 2010;50 Suppl 1:S74-9. doi: 10.1016/j.ypmed.2009.07.027 19751757

[50] Nichani V, Vena JE, Friedenreich CM, Christie C, McCormack GR. A population-based study of the associations between neighbourhood walkability and different types of physical activity in Canadian men and women. Prev Med. 2019;129:105864. doi: 10.1016/j.ypmed.2019.105864 31654728

[51] Dixon BN, Ugwoaba UA, Brockmann AN, Ross KM. Associations between the built environment and dietary intake, physical activity, and obesity: A scoping review of reviews. Obes Rev. 2021;22(4):e13171. doi: 10.1111/obr.13171 33369097 PMC8629168

[52] Kärmeniemi M, Lankila T, Ikäheimo T, Koivumaa-Honkanen H, Korpelainen R. The built environment as a determinant of physical activity: a systematic review of longitudinal studies and natural experiments. Ann Behav Med. 2018;52(3):239–51. doi: 10.1093/abm/kax043 29538664

[53] Smith M, Hosking J, Woodward A, Witten K, MacMillan A, Field A, et al. Systematic literature review of built environment effects on physical activity and active transport - an update and new findings on health equity. Int J Behav Nutr Phys Act. 2017;14(1):158. doi: 10.1186/s12966-017-0613-9 29145884 PMC5693449

[54] Pineo H, Zimmermann N, Davies M. Integrating health into the complex urban planning policy and decision-making context: a systems thinking analysis. Palgrave Commun. 2020;6(1):21. doi: 10.1057/s41599-020-0398-3

[55] Conderino SE, Feldman JM, Spoer B, Gourevitch MN, Thorpe LE. Social and economic differences in neighborhood walkability across 500 U.S. Cities. Am J Prev Med. 2021;61(3):394–401. doi: 10.1016/j.amepre.2021.03.014 34108111

[56] Bibri SE, Krogstie J, Kärrholm M. Compact city planning and development: Emerging practices and strategies for achieving the goals of sustainability. Developments in the Built Environment. 2020;4:100021. doi: 10.1016/j.dibe.2020.100021

[57] Jeong I, Choi M, Kwak J, Ku D, Lee S. A comprehensive walkability evaluation system for promoting environmental benefits. Sci Rep. 2023;13(1):16183. doi: 10.1038/s41598-023-43261-0 37758828 PMC10533864

[58] Moreno C, Allam Z, Chabaud D, Gall C, Pratlong F. Introducing the “15-minute city”: sustainability, resilience and place identity in future post-pandemic cities. Smart Cities. 2021;4(1):93–111. doi: 10.3390/smartcities4010006

[59] Allam Z, Nieuwenhuijsen M, Chabaud D, Moreno C. The 15-minute city offers a new framework for sustainability, liveability, and health. Lancet Planet Health. 2022;6(3):e181–3. doi: 10.1016/S2542-5196(22)00014-6 35278381

[60] Andrade AC de S, Mingoti SA, Fernandes AP, Andrade RG de, Friche AA de L, Xavier CC, et al. Neighborhood-based physical activity differences: Evaluation of the effect of health promotion program. PLoS One. 2018;13(2):e0192115. doi: 10.1371/journal.pone.0192115 29401506 PMC5798787

[61] Fox EH, Chapman JE, Moland AM, Alfonsin NE, Frank LD, Sallis JF, et al. International evaluation of the Microscale Audit of Pedestrian Streetscapes (MAPS) Global instrument: comparative assessment between local and remote online observers. Int J Behav Nutr Phys Act. 2021;18(1):84. doi: 10.1186/s12966-021-01146-3 34193160 PMC8247070

[62] Magalhães AS, Andrade AC de S, Moreira B de S, Lopes AADS, Caiaffa WT. Physical and social neighborhood disorder in Latin American cities: a scoping review. Cad Saude Publica. 2023;39(9):e00038423. doi: 10.1590/0102-311XPT038423 37729304 PMC10513154

[63] Vegi ASF, Fernandes Filho EI, Pessoa MC, Ramos KL, Ribeiro AQ. Walkability and healthy aging: an analytical proposal for small and medium-sized Brazilian cities. Cad Saude Publica. 2020;36(3):e00215218. doi: 10.1590/0102-311x00215218 32187294

[64] de Magalhães DJAV, Rigatto IB. Individual perceptions of critical factors on route affecting the willingness of direct commuting trips by bicycle in a hilly city. Transportation. 2023;52(1):127–53. doi: 10.1007/s11116-023-10414-z

[65] Souza RCF de, Oliveira VB de, Pereira DB, Costa HS de M, Caiaffa WT. Viver próximo à saúde em Belo Horizonte. Cad Metrop. 2016;18(36):326–44. doi: 10.1590/2236-9996.2016-3601

[66] Frank LD, Wali B. Monitoring changes in walkability over time:a framework for assessing impacts on social disparities, greenhouse gas emissions, and public health. SSRN Electron J. 2021; 33. 10.2139/ssrn.4551261

[67] Badland H, White M, Macaulay G, Eagleson S, Mavoa S, Pettit C, et al. Using simple agent-based modeling to inform and enhance neighborhood walkability. Int J Health Geogr. 2013;12:58. doi: 10.1186/1476-072X-12-58 24330721 PMC3874648

[68] Pontin FL, Jenneson VL, Morris MA, Clarke GP, Lomax NM. Objectively measuring the association between the built environment and physical activity: a systematic review and reporting framework. Int J Behav Nutr Phys Act. 2022;19(1):119. doi: 10.1186/s12966-022-01352-7 36104757 PMC9476279

