# Supplementary material for: What is the cross-sectional association of geospatially derived walkability with walking for leisure and transport?
Source: PLoS One. 2025 Mar 21;20(3):e0320202. doi: 10.1371/journal.pone.0320202 (PMC11927896; doi:10.1371/journal.pone.0320202)
Supplement: Supporting Materials — (PDF) [file pone.0320202.s001.pdf]

What is the cross-sectional association of geospatially derived walkability with walking for leisure and transport?

Adalberto A. S. Lopes[1], Larissa L. Lima[2], Amanda S. Magalhães[1], Amanda C. S. Andrade[1], Tiago Canelas[3], Louise Foley[3], Tolu Oni[3], Waleska T. Caiaffa[1].

[1] Observatory for Urban Health in Belo Horizonte, Federal University of Minas Gerais, Brazil;
[2] Center for Modeling Social Systems, Norwegian Research Centre, Kristiansand, Norway;
[3] MRC Epidemiology Unit, University of Cambridge, England, United Kingdom.

-- Supplementary Material –

Through geospatial-based macroscale measures, walkability was created for the Belo Horizonte city. Using step-by-step procedures, several shapefiles' vectors were aggregated to compose the index (**C1**). Considering the census tract as the unit of measurement, the index was composed of three indicators: land use mix, intersection density, and net residential density [1]. Other geospatial-based variables, such as income *per capita* and land slope [2] were included once they may be relevant to city data interpretation (**F1**).

A robust statistical test was applied to identify the suitable analytical modelling according to the dataset characteristics and study design [3,4]. The first stage summarizes a descriptive as a prerequisite for processing the data. As the proportion of zeros is high, the sample size of those who do some walking for leisure and transportation is low. Moreover, the variance of the outcomes was much larger than the average, which may represent an issue in performing the Poisson regression model, for instance, once it indicates a possible over-dispersion. Often, a variable transformation is an alternative, even to apply an approximation to the normal distribution. The logarithm is known as the most popular proceeding and the square root the more complex, but as the results show, not even they seemed to be able to normalize or approximate the distribution, implying likely tricky findings interpretation on the original scale beyond being subjected to a bias (**F2**).

Generalized linear mixed models (GLMM) appear to be a safer path in this context. Thus, it was selected for the next stage as the suitable one. On the original observed and predicted values from various regression models with continuous positively skewed data, the GLM-Gamma seems a potential model. Still, due to the low sample size when working with only non-zero cases, this alternative was discarded (**F3**). The standard error from the Poisson regression model is much smaller than those of the other GLMs, confirming an over-dispersion. Furthermore, just one factor is insufficient for selecting an appropriate model. Hence Akaike Information Criterion and dispersion parameter were used to select the analytical approach (**T1**). Finally, the complete values of the Incidence Rate Ratio and the 95% Confidence Interval, used in the main analysis, are presented (**T2**).

In addition to taking into account the statistical modelling, based on a brief literature review ecological model [5] a DAG – directed acyclic graph [6–8] was drawn to establish the conceptual modelling to identify the minimal sufficient covariates (grouped into three blocks) needed to build the adjustment sets for estimating the total effect of the walkability index on general walking outcome (**F4**).

Supplement C1. **Chart 1.** Description of the geospatial-based macroscale indicators and procedures adopted to create the walkability index in Belo Horizonte, Brazil.

Indicator	Vectoral composition	Source, year	Procedures adopted	Product and interpretation
**Land use mix**†	Plot area polygons with general description of land use;<br>+ Aggregated points or centroids of polygons referring to previously uncaptured land use;<br>+ Identification points of official commercial licenses registered for each establishment and/or service.	BHMap, 2020<br>BHMap, 2020<br>CNAE, 2020	a. The plots were classified into 8 categories (i.e.: residential, retail, entertainment, food-related, civic/institutional, office, public and private recreation)<br>b. Relevant land use points and/or centroids that had not yet been considered before (i.e.: parks, plazas, green/blue areas, sports centres, health academy poles, and others) were intersected to the plots, adding them to the classification pre-established;<br>c. The same procedure was adopted for the 1,085 points containing commercial license identifications;<br>d. Based on the census tract, the areas of the plots were intersected and summarized to compute the amount of each land use that composes them;<br>e. The entropy was calculated from the formula§:<br>$Entropy = -\frac{\sum k(p_k 1n\, p_k)}{1n\, N}$	Based on a theory concept [9] that ranges from zero [predominance of only one type of land use] to one [equal distribution among all categories of use].
**Intersection density**†	Points originally created through the vertices of the road network lines.	Open street map, 2023	a. The nodes that connect street segments were identified to create vertices;<br>b. A 10-meters Euclidean buffer around those vertices was created to avoid overestimating the connections;<br>c. The overlapping buffers were dissolved;<br>d. A centroid of each buffer was attributed;<br>e. Selected only those formed by three or more street segments.	Ratio between the number of street connection, formed by three or more street segments, and the census tract total area (transforming in z-score).
**Net residential density**†	Plot area polygons with general description of residential land use;	BHMap, 2020	From the plots classified as residential use, their areas were used to compute the amount of residential territory (i.e.: housing, apartment, duplex, rental dwelling) that make up the area of the census tract from IBGE.	Ratio of the residential land use divided by the census tracts area (transforming in z-score).
**Walkability**	Polygons (census tract)	Frank *et al*., 2010 [1].	Composed of the three indicators, using the formula:<br>$WI = (Entropy) + (2 * ID_{zs}) + (NRD_{zs})$	Continuous index for the whole city, ranging from -5.37 to 175.69, where larger values represent higher level of walkability.

Note: *The Spatial Research Data Infrastructure (SRDI) originally used is European Petroleum Survey Group (EPSG) projection 31983, SIRGAS 2000/UTM 23S.*

BHMap: *Municipality of Belo Horizonte project which provides a set of open maps/tools for general use;* ***IBGE***: *Brazilian Institute of Geography and Statistics (census data from 2010);* ***CNAE***: *National Classification of Economic Activities;* ***†***: *All with measure and analysis scale of quantitative ratio;* ***§***: *where, "****p****" is the proportion of land use, "****N****" is the number of land use categories and "****k****" is the land use category;*

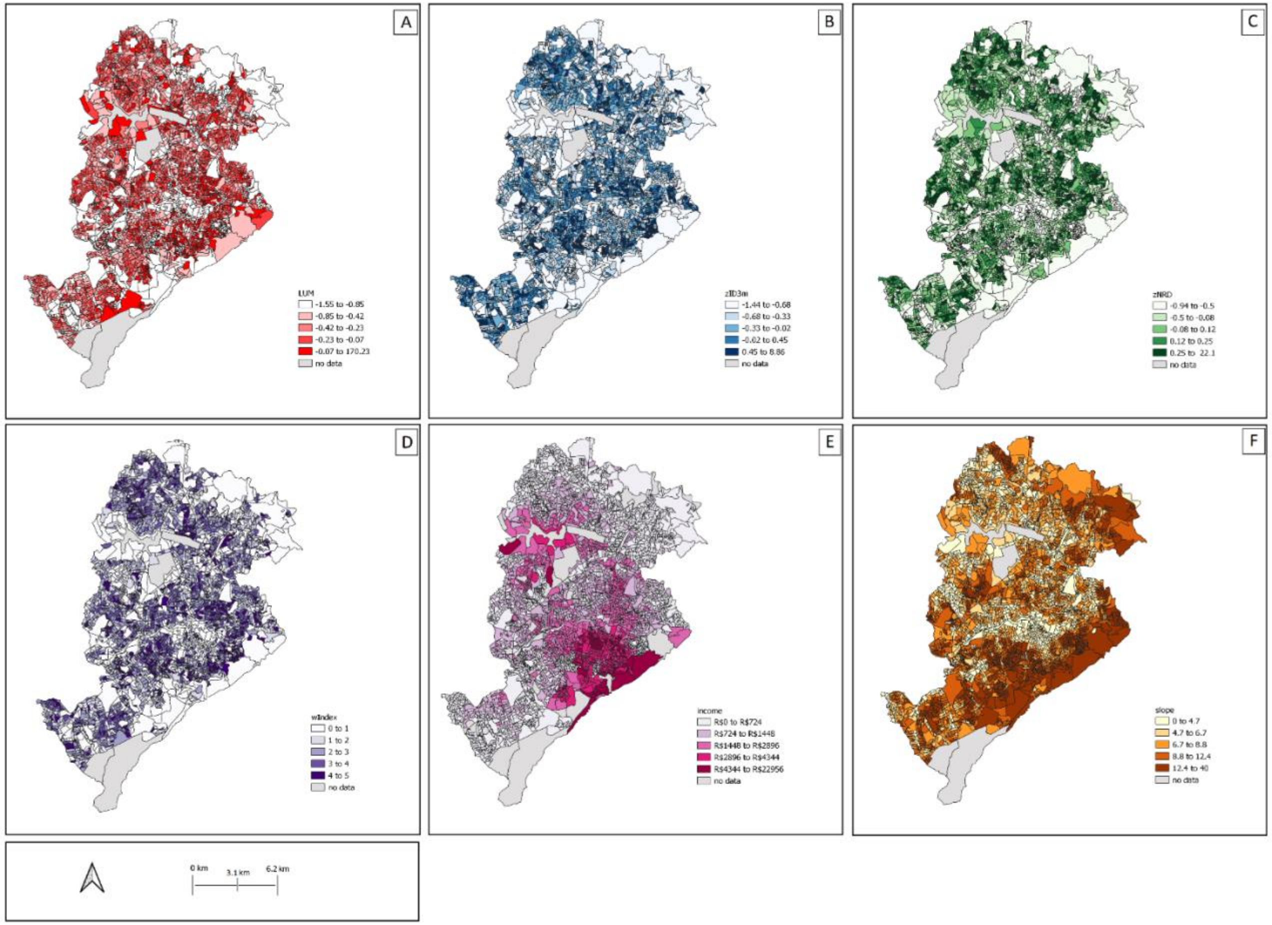

LUM: *Land use mix (entropy);* ***zID3m***: *Intersection density by three or more street segments (z-score);* ***zNDR***: *Net residential density (z-score);* ***wIndex***: *Walkability index developed according to Frank (2010);* ***Income***: *Contextual income in Brazilian Real (R$);* ***Slope***: *Percentage of land slope (%).*

Supplement F1. **Figure 1.** Geospatial-based macroscale indicators use on walkability index creation and city interpretation relevant factors in Belo Horizonte, Brazil. (n=1,372)

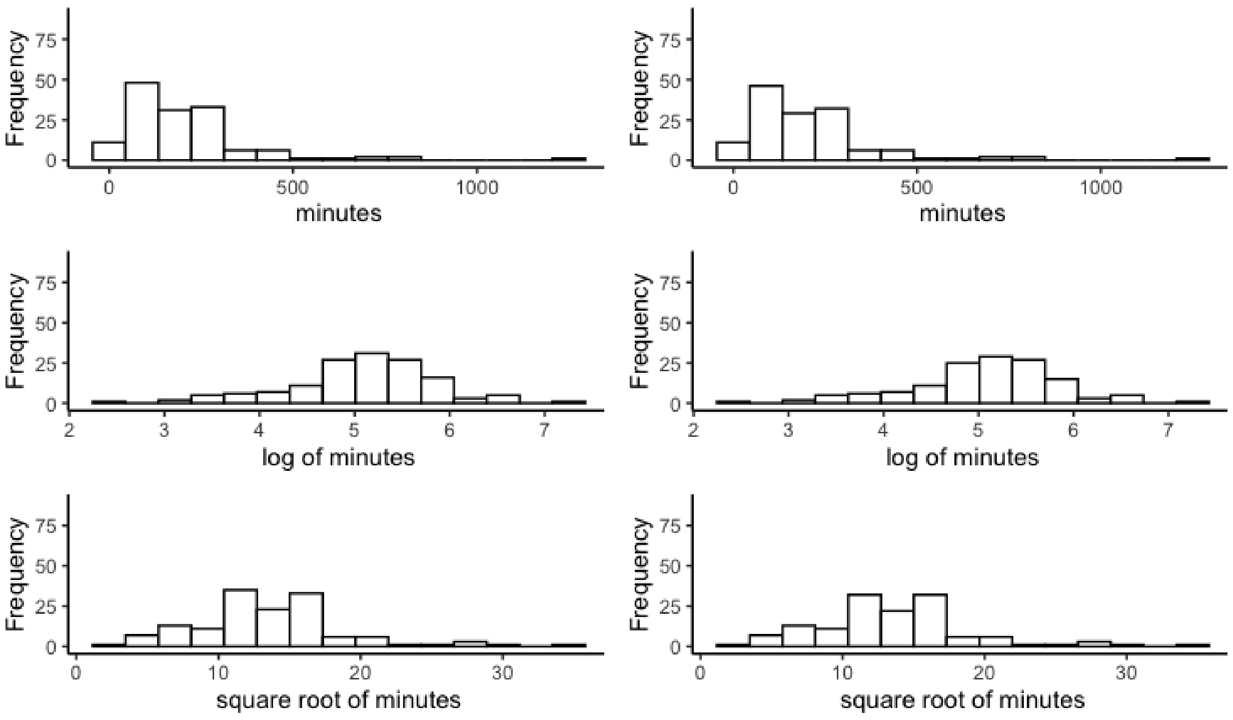

Walking for Leisure
(n=142 active)

Walking for Transportation
(n=137 active)

log: *Logarithm (most popular transformations for a non-normal continuous outcome);* ***sqrt***: *Square root (more complex transformations for a non-normal continuous outcome);*

Supplement F2. **Figure 2**. Distribution of the original and transformed outcomes of walking (leisure/transportation) in Belo Horizonte, Brazil. (n=1,372)

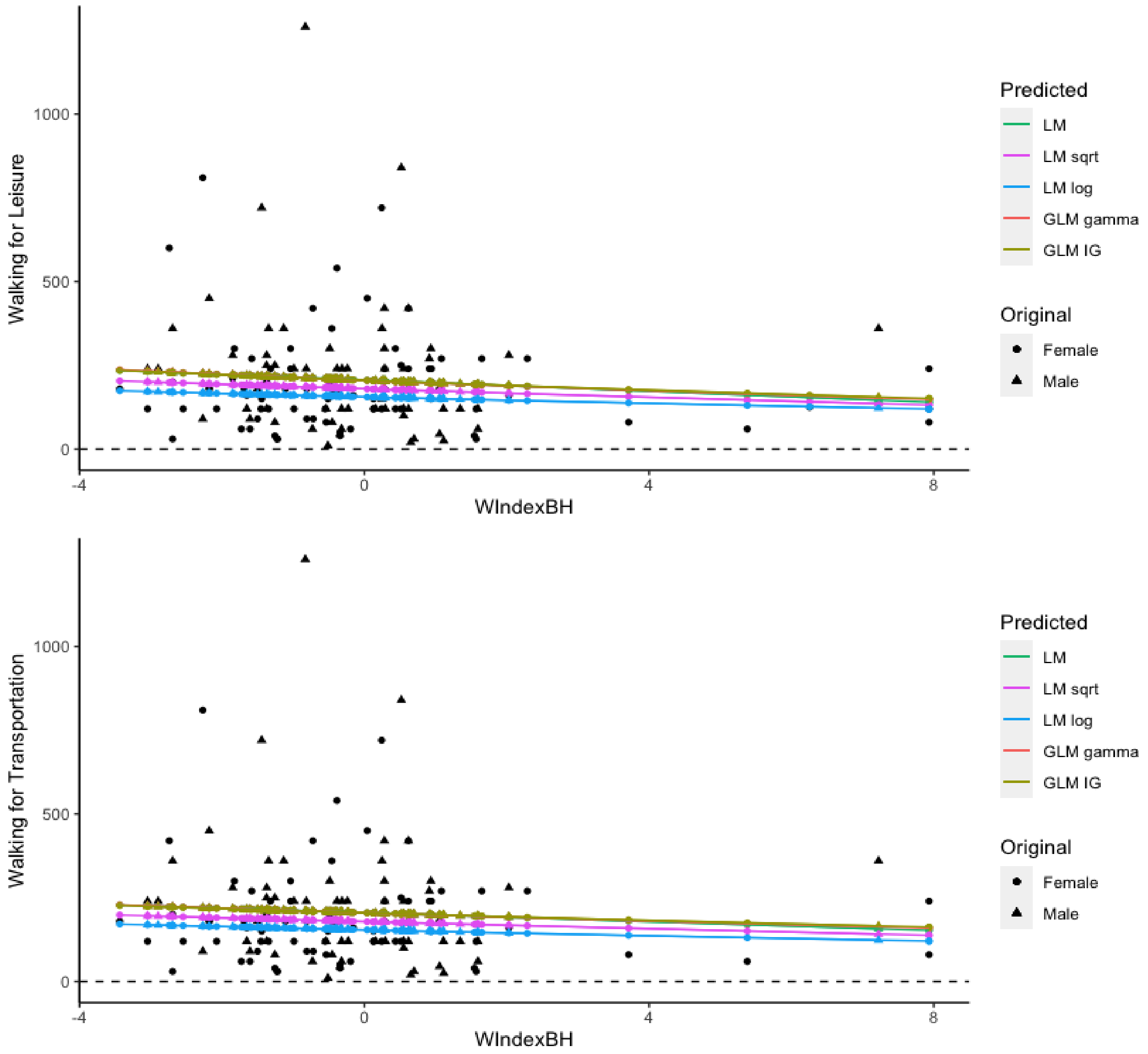

***WIndexBH**: Walkability index from census tract in Belo Horizonte, Brazil; **LM**: Standardized linear regression model; **log**: Logarithm (most popular transformations for a non-normal continuous outcome); **sqrt**: Square root (more complex transformations for a non-normal continuous outcome); **GLM**: Generalized linear model; **IG**: Inverse Gaussian.*

Supplement F3. **Figure 3**. Observed (original) and predicted values from various regression models with continuous positively skewed data of walking (leisure/transportation) and walkability index in Belo Horizonte, Brazil. (n=1,372)

Supplement T1. **Table 1**. Estimated associations between walking (leisure/transportation) and walkability index, using four regression models to identify the more appropriate according to the count data characteristic. Belo Horizonte, Brazil. (n=1,372)

		**Regression models**			
		**LM**	**GLM-Poisson**	**GLM-QPoisson**	**GLMM-NB**
**Walking for leisure**					
Intercept	Estimate	17.889	1.862	2.881	1.875
	SE	2.095	0.908	0.117	0.908
	p-value	<0.001	0.040	<0.001	0.039
	$CI_{95\%}$	13.779 – 22.004	17.830 – 17.861	2.642 – 3.103	0.094 – 3.656
Walkability index	Estimate	-0.528	0.010	-0.031	0.033
	SE	0.970	0.000	0.057	0.006
	p-value	0.586	<0.001	0.586	<0.001
	$CI_{95\%}$	-2.432 – 1.375	0.970 – 0.981	-0.153 – 0.073	0.021– 0.045
	AIC	17,005.99	41,676,875	4,347.78	829,461.50
	DP	-	-	115,755.20	0.0139
**Walking for transportation**					
Intercept	Estimate	17.415	1.823	2.853	1.832
	SE	2.071	0.905	0.119	0.906
	p-value	<0.001	0.044	<0.001	0.043
	$CI_{95\%}$	13.352 – 21.478	0.047 – 3.598	2.609 – 3.078	0.056 – 3.608
Walkability index	Estimate	-0.645	0.003	-0.040	0.021
	SE	0.959	0.000	0.059	0.006
	p-value	0.501	<0.001	0.501	<0.001
	$CI_{95\%}$	-2.527 – 1.235	2.348 – 5.198	-0.165 – 0.067	0.008 – 0.033
	AIC	16,973.97	40,810,356	3,806.72	811,560.20
	DP	-	-	116,271.40	0.0136

LM: *Standardized linear regression model;* ***GLM:*** *Generalized linear model****;*** ***GLM-Poisson****: Poisson regression;* ***GLM-QPoisson****: Quasi-Poisson regression;* ***GLMM-NB****: Negative Binomial regression;* ***SE****: standard error;* ***$CI_{95\%}$****: Confidence interval;* ***AIC****: Akaike Information Criterion;* ***DP****: dispersion parameter.*

Supplement T2. Table 2. Multilevel negative binomial regression models of walkability index & walking for leisure and transportation in Belo Horizonte, Brazil. (n=1,372).

**Models**	**Physical activity domain**	
	Walking for Leisure	Walking for Transportation
	**IRR** ($CI_{95\%}$)	**IRR** ($CI_{95\%}$)
Walkability index		
Model 0	1.03 (1.02-1.05)*	1.02 (1.01-1.03)*
Model 1	1.01 (0.99-1.02)	0.97 (0.96-0.98)
Model 2	1.15 (1.13-1.16)*	1.13 (1.11-1.15)*
Model 3	1.06 (1.05-1.08)*	1.05 (1.04-1.06)*
Model 4	1.03 (1.02-1.04)*	1.02 (1.01-1.04)*
Model 5	1.33 (1.32-1.35)*	1.22 (1.20-1.24)*

***IRR**: Incidence rate ratio; **$CI_{95\%}$**: Confidence interval; *: Statistically significant, kept $p<0.001$; **Model 0**: Crude analysis; **Model 1**: Adjusted by individual sociodemographic; **Model 2**: Adjusted by health indicators; **Model 3**: Adjusted by neighbourhood contextual; **Model 4**: Adjusted by the interaction of income and land slope, geospatially derived urban macroscale measures (census tract); **Model 5**: Models of 1 to 4 combined.*

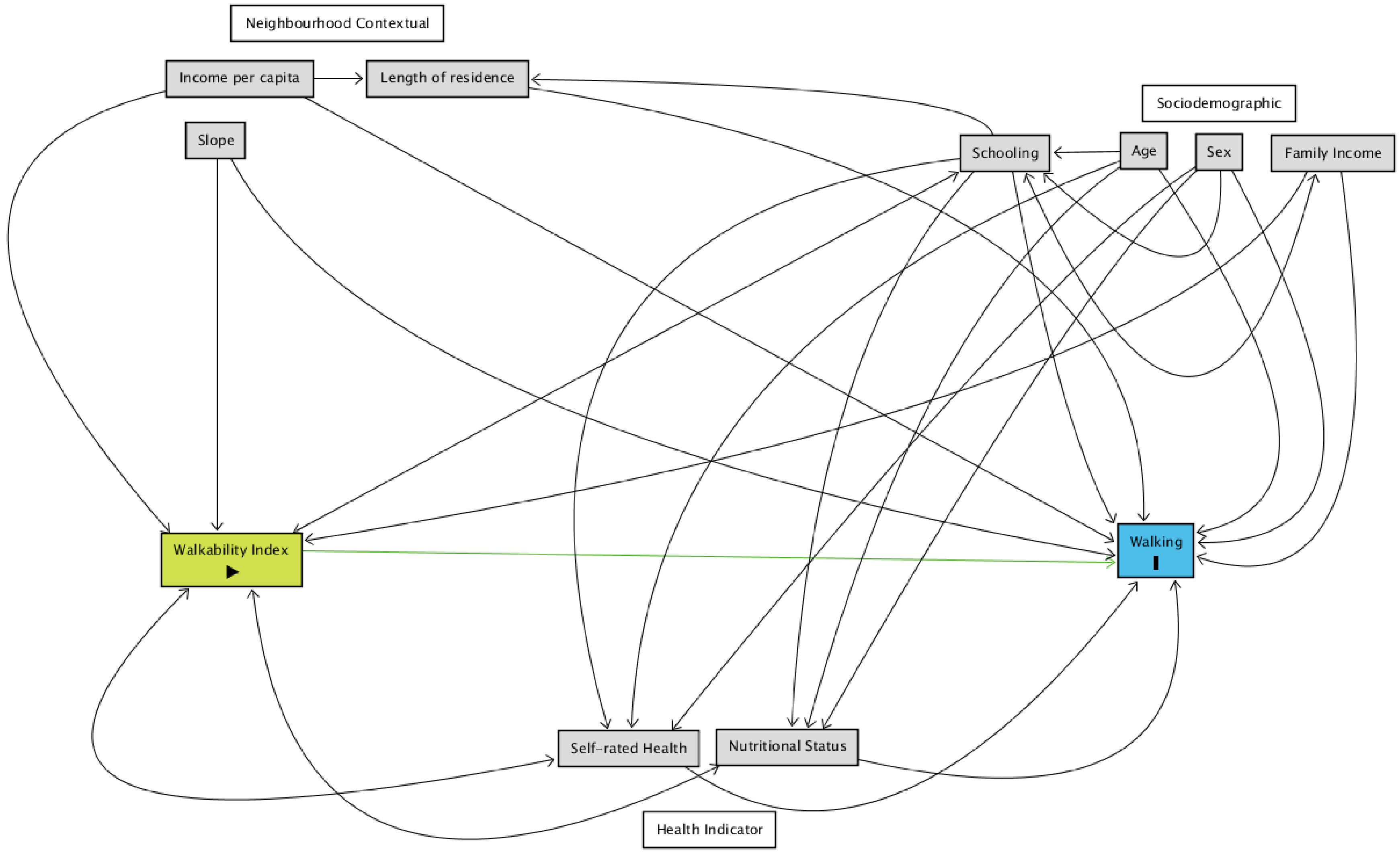

Supplement F4. **Figure 4**. Directed acyclic graph (DAG) shows the conceptual model to minimal sufficient adjustment sets for estimating the total effect of the walkability index on walking (leisure/transportation) in Belo Horizonte, Brazil.

References

1. Frank LD, Sallis JF, Saelens BE, Leary L, Cain K, Conway TL, et al. The development of a walkability index: application to the Neighborhood Quality of Life Study. Br J Sports Med. 2010;44: 924–933. doi:10.1136/bjsm.2009.058701
2. Souza RCF de, Oliveira VB de, Pereira DB, Costa HS de M, Caiaffa WT. Living near health at Belo Horizonte. Cad Metrópole. 2016;18: 326–344. doi:10.1590/2236-9996.2016-3601
3. Lamb KE, White SR. Categorisation of built environment characteristics: the trouble with tertiles. Int J Behav Nutr Phys Act. 2015;12: 19. doi:10.1186/s12966-015-0181-9
4. Akram M, Cerin E, Lamb KE, White SR. Modelling count, bounded and skewed continuous outcomes in physical activity research: beyond linear regression models. Int J Behav Nutr Phys Act. 2023;20: 57. doi:10.1186/s12966-023-01460-y
5. Bauman AE, Reis RS, Sallis JF, Wells JC, Loos RJF, Martin BW, et al. Correlates of physical activity: why are some people physically active and others not? Lancet (London, England). 2012;380: 258–71. doi:10.1016/S0140-6736(12)60735-1
6. Ferguson KD, McCann M, Katikireddi SV, Thomson H, Green MJ, Smith DJ, et al. Evidence synthesis for constructing directed acyclic graphs (ESC-DAGs): A novel and systematic method for building directed acyclic graphs. Int J Epidemiol. 2020;49: 322–329. doi:10.1093/ije/dyz150
7. Tennant PWG, Murray EJ, Arnold KF, Berrie L, Fox MP, Gadd SC, et al. Use of directed acyclic graphs (DAGs) to identify confounders in applied health research: review and recommendations. Int J Epidemiol. 2021;50: 620–632. doi:10.1093/ije/dyaa213
8. Digitale JC, Martin JN, Glymour MM. Tutorial on directed acyclic graphs. J Clin Epidemiol. 2022;142: 264–267. doi:10.1016/j.jclinepi.2021.08.001
9. Shannon CE. A Mathematical Theory of Communication. Bell Syst Tech J. 1948;27: 379–423. doi:10.1002/j.1538-7305.1948.tb01338.x
